# Development of atrial fibrillation in patients with heart failure and vice versa: incidence, risk factors, and their impact on survival

**DOI:** 10.1093/eschf/xvag152

**Published:** 2026-05-28

**Authors:** Arietje J L Zandijk, Hailun Qin, Jasper Tromp, Michiel Rienstra, Adriaan A Voors

**Affiliations:** Department of Cardiology, University of Groningen, University Medical Center Groningen, Hanzeplein 1, Groningen 9713 GZ, The Netherlands; Department of Cardiology, University of Groningen, University Medical Center Groningen, Hanzeplein 1, Groningen 9713 GZ, The Netherlands; Department of Cardiology, University of Groningen, University Medical Center Groningen, Groningen, The Netherlands; Saw Swee Hock School of Public Health, National University of Singapore and the National University Health System, Singapore, Singapore; National Heart Centre Singapore and Duke-NUS Medical School, Singapore, Singapore; Department of Cardiology, University of Groningen, University Medical Center Groningen, Hanzeplein 1, Groningen 9713 GZ, The Netherlands; Department of Cardiology, University of Groningen, University Medical Center Groningen, Hanzeplein 1, Groningen 9713 GZ, The Netherlands

**Keywords:** Atrial fibrillation, Heart failure, Mortality, Temporality

## Abstract

**Introduction:**

Atrial fibrillation (AF) and heart failure (HF) frequently coexist, and their combination is associated with poorer outcomes. The bidirectional risk and the impact of which disease develops first on mortality remain unclear.

**Methods:**

We studied 31 374 UK Biobank participants with new-onset AF or HF (2006–22). Multivariable Cox models assessed risk factors, incidence, and mortality risk.

**Results:**

The risk of developing HF was higher in patients with AF than in those without AF (45.1 vs 3.87 per 1000 person-years; aHR 3.15, 95% CI 3.0–3.31). The risk of developing AF in patients with HF was higher than in those without HF (25.4 vs 2.27 per 1000 person-years; aHR 2.75, 95% CI 2.62–2.90). HF patients had a 31% higher risk of subsequent AF, compared with the risk of AF patients developing HF (*P* < .05). Mortality risk was substantially higher in patients with both conditions. AF before HF conferred a nearly four-fold increased risk (aHR 3.8, 95% CI 3.5–4.2), while HF before AF more than doubled risk (aHR 2.0, 95% CI 2.1–2.6), compared with either condition alone. Regardless of which disease was diagnosed first, there was no significant difference in mortality risk (aHR 0.95, 95% CI 0.85–1.07).

**Conclusions:**

AF and HF strongly predispose to each other, with HF conferring a higher relative risk for incident AF. The coexistence of both diseases substantially increases mortality regardless of which disease developed first, emphasizing the need for strategies to prevent the development of HF in patients with AF and vice versa.

## Graphical Abstract

**Graphical Abstract:**
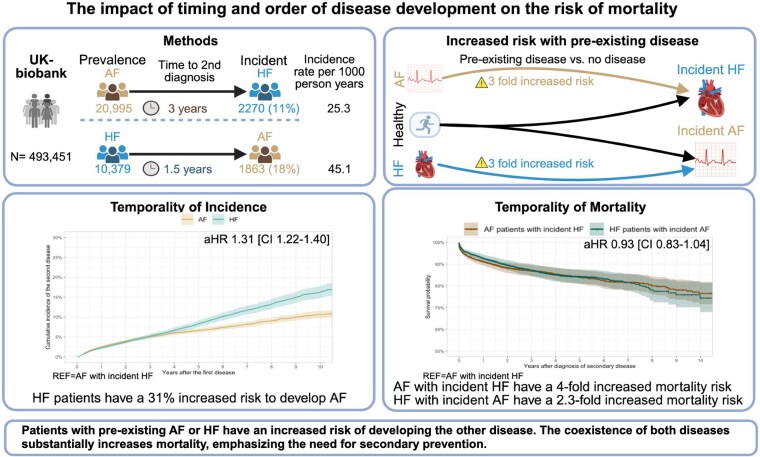

Patients with pre-existing AF or HF have an increased risk of developing the other disease. The coexistence of both diseases substantially increases mortality, emphasizing the need for secondary prevention.

## Introduction

Atrial fibrillation (AF) and heart failure (HF) represent two of the most common cardiovascular epidemics, each affecting 1%–3% of the global population, with prevalence projected to triple over the next two decades.^1–3^ These conditions frequently coexist: AF affects 25%–50% of patients with clinically overt HF, while approximately one-third of AF patients develop HF.^4–7^ Their co-occurrence substantially increases the risk of stroke, HF exacerbations, hospitalizations, and all-cause mortality, while diminishing quality of life and escalating healthcare burden.^1,2^

Previous studies have established the augmented risk associated with AF and HF.^8^ However, most of these studies were conducted in hospital-based populations and did not systematically evaluate how the temporal sequence of disease development impacts long-term outcomes. Specifically, whether developing AF before HF, or vice versa, differentially affects mortality risk remains unclear. Understanding this temporal relationship has important implications. If the coexisting diseases represent different phenotypic expressions of the same underlying pathophysiology, the order of disease diagnosis may not affect outcomes.

Therefore, the objective of the present study was (i) to evaluate the risk factors, incidence, and mortality risk for developing HF in AF patients and AF in HF patients; (ii) to directly compare the relative risks of developing the secondary condition; and (iii) to evaluate whether the temporal sequence of disease development impacts long-term all-cause mortality.

## Methods

### Database

United Kingdom (UK) Biobank (2006–22) is a longitudinal, prospective, community-based biomedical cohort of >500 000 adults aged 40–69 years at 22 centres. Detailed descriptions of the database and data collection have been previously published.^9,10^ In summary, the UK Biobank aimed to recruit a diverse cohort representative of various socioeconomic backgrounds, ethnicities, and rural and urban residencies. Extensive phenotypic data, functional and physical measurements, and biological samples were collected at baseline. Data on race, smoking status, and socioeconomic data were also collected. To capture clinical outcomes during follow-up, participants’ records were linked to primary care, inpatient and outpatient hospital records, and cancer and death registrations. Ethics approval was granted by the local ethics committee and all participants consented electronically.^9,10^

### Cohort creation

**Figure 1:**
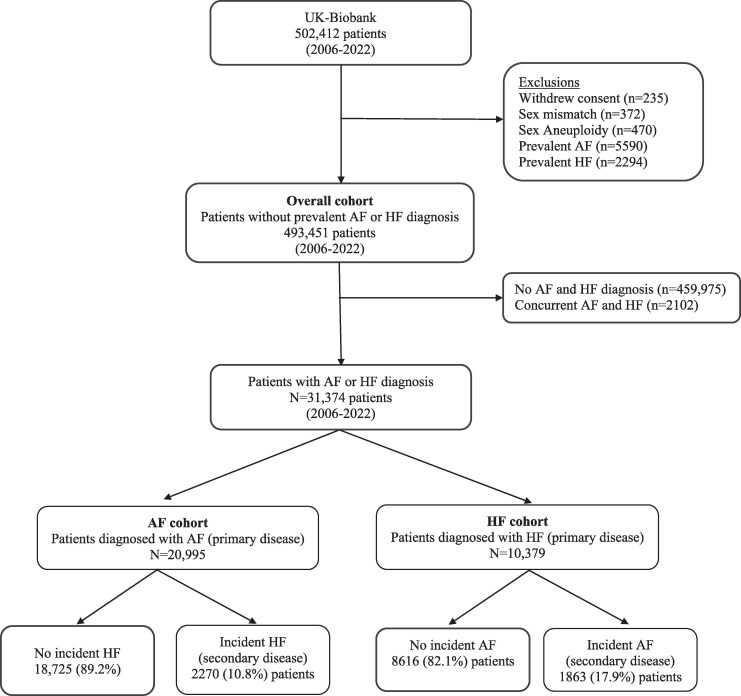
Flowchart participants UK Biobank

Cohort creation is illustrated in *Figure 1*. To address the study objectives, three cohorts were created based on the incidence of AF or HF diagnosis. The overall cohort (*N* = 493 451) consisted of all participants who did not have a prevalent diagnosis of AF or HF at baseline (i.e. UK Biobank cohort entry; *Figure 1*).

To assess the risk for the development of a secondary cardiovascular disease (either AF or HF) and mortality, an independent disease-specific AF cohort (*N* = 20 955) and HF cohort (*N* = 10 379) were created. Inclusion into the AF or HF cohorts was based on the primary disease diagnosis. The primary disease was defined as the disease, AF or HF, with the earliest calendar year date of diagnosis, regardless of the development of the other disease at a later calendar date. Patients were included in the disease-specific cohort on the date of the primary disease diagnosis (i.e. baseline or time zero) and followed till diagnosis of the secondary cardiovascular disease (i.e. AF or HF), death, or the end of cohort follow-up (31 October 2022 for England, 31 May 2022 for Wales, and 31 August 2022 for Scotland). At baseline, all patients in the disease-specific cohorts were only diagnosed with a single disease, either AF or HF. Patients who were diagnosed with both AF and HF within a 30-day window were excluded (*n* = 2102).^4^

### Disease definitions

Previously validated definitions of AF and HF in the UK Biobank were used to ascertain disease diagnoses.^11,12^ A diagnosis of AF was determined by self-reported questionnaires (20002[1471], 20002[1483]) and inpatient hospital records (ICD-9 code: 4273; ICD-10 code: I48; OPCS4:K621, K622, K623; Supplementary Table S1).^11^

Likewise, HF was also captured from self-reported questionnaires (nurse interview and questionnaires: 20002[1076], 20004[1098]) and inpatient hospital records (ICD-9 code: 428; ICD-10 codes: I50, I110, I130, I132, Z941, T862; OPCS4: K02; Supplementary Table S1).^13^ The date and cause for hospital admission were obtained by linkage of records from Hospital Episode Statistics for England, Patient Episode Database for Wales, and Scottish Morbidity Records.

### Covariates

Comorbidities, such as hypertension, hyperlipidaemia, diabetes mellitus type 2, myocardial infarction, chronic obstructive pulmonary disease, valvular disease, stroke, and chronic kidney disease (CKD), were identified from self-reported medical history, medication, and in-hospital records. The diagnosis of diabetes mellitus type 2 at baseline was defined by integration of multiple data sources, coupled with a random glucose level ≥ 11.1 mmol/L or glycated haemoglobin (HbA1c) level ≥ 48 mmol/mol (6.5%) at first assessment.^14,15^ Obesity was determined as a body mass index ≥ 30 kg/m^2^. Alcohol consumption and smoking status were derived from questionnaires and defined as current, former, and never.

The socioeconomic status was calculated by the Townsend deprivation index and categorized into three levels using national cut-off values of high socioeconomic status (<−2.08), medium socioeconomic status (−2.08–1.40), and low socioeconomic status (≥1.40).^16^ Medication use was captured from self-reported questionnaires and included angiotensin-converting enzyme inhibitors, angiotensin-receptor blockers, beta-blockers, calcium channel blockers, and lipid-lowering drugs (Supplementary Table S1).

### Statistical analysis

Descriptive data were presented as frequencies and percentages or mean and standard deviation, as appropriate. Continuous variables with a non-normal distribution were presented as median and interquartile range. The assumption of normality was determined from Q–Q plots. Baseline characteristics were compared with the Student’s independent *t*-test, Mann–Whitney, and *χ*² test, as appropriate.

Crude incidence rates for all outcomes were calculated per 1000 person-years. Mortality risk was compared between patients with a primary disease of AF or HF with multivariable Cox proportional hazards models. Only patients diagnosed with both AF and HF were included. They were followed from the calendar date of the diagnosis for the secondary disease (i.e. time 0) till death or end of cohort follow-up, whichever was first. Additional Cox models were performed within disease-specific cohorts to evaluate the augmented risk due to the presence of both diseases compared with a single disease, AF or HF, with the use of binary time-varying exposure variables to prevent immortal time bias. All patients were followed from the date of primary disease diagnosis. A patient contributed unexposed time (only had the primary disease) till the date of the secondary disease diagnosis, in which a patient contributed exposed time from the date of secondary disease diagnosis till the end of follow-up or death. A patient who did not acquire a secondary disease was considered unexposed for the entire follow-up period.

For non-fatal outcomes, including primary and secondary disease diagnosis, the Fine and Gray extension of the Cox model was used to account for the competing risk of death. We compared the risk of developing AF or HF in patients with one disease, compared with those with no existing disease, using appropriate adjustments. All Cox models were adjusted for time-fixed (e.g. age, sex, obesity, current smoker) and time-updated clinically significant confounders, identified *a priori*. Time-updated comorbidities included hypertension, hyperlipidaemia, diabetes mellitus type II, myocardial infarction, chronic obstructive pulmonary disease, valvular disease, stroke, and CKD. Kaplan–Meier curves and cumulative incidence functions were created, as appropriate.

Risk factors for outcomes were identified as statistically significant predictors in multivariable Cox models (*P* < .05). Potential heterogeneity in effect based on age and sex was also investigated with the inclusion of interaction terms. A two-sided alpha of <0.05 was considered statistically significant. All statistical analyses were performed in RStudio (version 4.0.1, R Foundation for Statistical Computing, Vienna, Austria).^17^

## Results

### Incidence of primary disease

Of the 502 412 patients enrolled in the UK Biobank (2006–22), 493 451 patients did not have prevalent AF or HF at baseline (overall cohort; *Figure 1*). Patients in the overall cohort had a median age of 58 (IQR: 50–63) years, and 55% were females (*Table 1*). Patient characteristics of HF with incident AF vs AF with incident HF can be found in Supplementary Table S2.

**Table 1:** Patient characteristics

Patient characteristics^a,b^	Overall cohort (*N* = 493 451)	AF cohort (*N* = 20 995)		HF cohort (*N* = 10 379)	
		No incident HF (*N* = 18 725)	Incident HF (*n* = 2270)	No incident AF (*N* = 8516)	Incident AF (*n* = 1863)
Age, years, median [IQR]	58.00 [50.00, 63.00]	71.57 [66.61, 75.58]	71.39 [67.31, 75.24]	69.81 [64.60, 74.18]	69.83 [65.36, 73.51]
Female	270464 (54.8)	7493 (40.0)	816 (35.9)^c^	3338(39.2)	615 (33.0)^c^
Caucasian	463987 (94.6)	18202 (97.8)	2189 (97.2)	7918 (93.7)	1750 (94.7)^c^
BMI, kg/m^2^ (mean ± SD)	27.40 ± 4.78	28.63 ± 5.12	30.08 ± 5.96^c^	29.49 ± 5.77	30.85 ± 5.99^c^
Obesity	118946 (24.3)	6152 (33.1)	956 (42.6)^c^	3335 (39.8)	901 (49.2)^c^
Waist to hip ratio (mean ± SD)	0.87 ± 0.09	0.90 ± 0.09	0.92 ± 0.09^c^	0.92 ± 0.09	0.94 ± 0.09^c^
Current smoker	52104 (10.6)	1722 (9.3)	286 (12.7)^c^	1528 (18.1)	260 (14.1)^c^
Current drinker	452484 (92.0)	17170 (92.0)	2041 (90.4)^c^	7302 (86.2)	1626 (87.8)
Socioeconomic status			^c^		
Low	107306 (21.8)	3973 (21.2)	582 (25.6)	2638 (31.0)	564 (30.3)
Median	135427 (27.5)	5015 (26.8)	625 (27.5)	2342 (27.5)	509 (27.4)
High	250103 (50.7)	9717 (51.9)	1063 (46.8)	3529 (41.5)	787 (42.3)
Medical history					
Diabetes mellitus 2	22869 (4.6)	2607 (13.9)	472 (20.8)^c^	2106 (24.7)	525 (28.2)^c^
Hypertension	150833 (30.6)	12287 (65.6)	1737 (76.5)^c^	6377 (74.9)	1532 (82.2)^c^
Hyperlipidaemia	100390 (20.4)	9268 (49.5)	1354 (59.6)^c^	5322 (62.5)	1230 (66.0)^c^
MI	10687 (2.2)	2029 (10.8)	383 (16.9)^c^	3076 (36.1)	613 (32.9)^c^
COPD	9456 (1.9)	1778 (9.5)	365 (16.1)^c^	1656 (19.4)	352 (18.9)
Stroke	8390 (1.7)	1996 (10.7)	264 (11.6)^c^	955 (11.2)	251 (13.5)^c^
Valvular disease	4640 (0.9)	2457 (13.1)	457 (20.1)^c^	1989 (23.4)	585 (31.4)^c^
CKD	1607 (0.3)	1464 (7.8)	269 (11.9)^c^	1304 (15.3)	296 (15.9)
Medication					
ACEi	48615 (9.9)	3343 (18.0)	589 (26.3)^c^	2204 (26.2)	605 (32.8)^c^
ARB	806 (0.2)	37 (0.2)	6 (0.3)	26 (0.3)	2 (0.1)
Beta-blockers	23702 (4.8)	1995 (10.7)	356 (15.7)^c^	1050 (12.4)	317 (17.1)^c^
Calcium channel blockers	33977 (6.9)	2553 (13.8)	520 (23.1)^c^	1530 (18.1)	475 (25.6)^c^
Lipid-lowering drug	41988 (8.5)	2743 (14.7)	541 (23.8)	1698 (20.0)	354 (19.0)

Abbreviations: ACEi, angiotensin-converting enzyme inhibitor; ARB, angiotensin-receptor blocker; BMI, body mass index; MI, myocardial infarction; COPD, chronic obstructive pulmonary disease; CKD, chronic kidney disease.

^a^Frequencies and percentages [*n* (%)] are presented, unless otherwise specified.

^b^All patient characteristics were reported at baseline or cohort entry. For the overall cohort, baseline was the start date for the UK Biobank. For the AF and HF disease-specific cohorts (i.e. primary disease), it was the date of diagnosis of the primary disease, except for BMI, obesity, waist to hip ratio, current smoker, current drinker, socioeconomic status, and medication.

^c^Depicts a statistically significant (*P* < 0.05) comparison between the overall disease-specific cohort (i.e. AF or HF cohort) and those within the disease-specific cohort who developed a secondary disease (incident HF or AF).

Over a median of 13.66 (IQR: 12.92–14.26) years of follow-up, 22 858 (4.63%) patients were diagnosed with AF and 12 649 (2.56%) were diagnosed with HF. The incidence rate of AF as the primary disease was 3.87 per 1000 person-years [incidence: 20 995 (4.25%) patients]. Heart failure was the primary disease diagnosis for 10 379 (2.10%) patients, with an incidence rate of 2.27 per 1000 person-years (first disease diagnosed; *Figure 1*).

### Atrial fibrillation cohort: incidence and risk factors for heart failure

**Figure 2:**
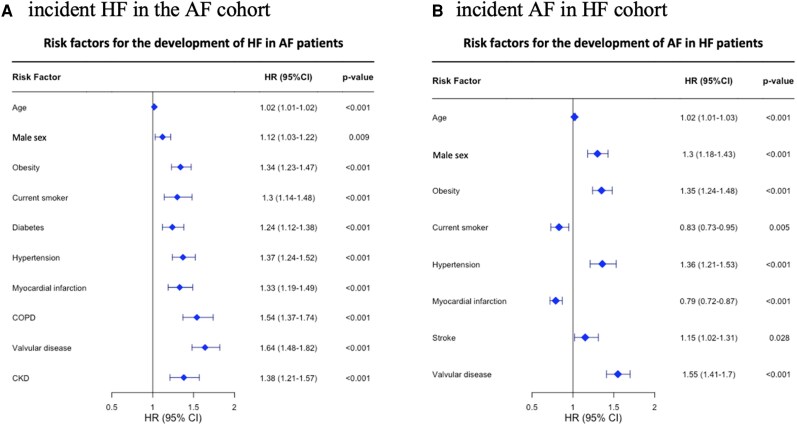
Risk factors for development of the secondary disease. (A) Incident heart failure in the atrial fibrillation cohort. (B) Incident atrial fibrillation in heart failure cohort

Of 20 995 patients with a primary disease diagnosis of AF, 2270 (10.81%) developed comorbid HF over a median 1.44 (IQR: 0.53–3.11) years of follow-up (incidence rate: 25.4 per 1000 person-years; *Table 2*). After multivariable adjustment, risk factors associated with incident HF among AF patients included advanced age, male sex, obesity, current smoking, diabetes mellitus type 2, hypertension, myocardial infarction, chronic obstructive pulmonary disease, valvular disease, and CKD (*Figure 2A*; *P* < .05 for all).

**Table 2:** Incidence of the secondary disease

	AF cohort (*N* = 20 995)	HF cohort (*N* = 10 379)
Risk of secondary disease diagnosis	Incident HF	Incident AF
Incidence, *n* (%)	2270 (10.81%)	1863 (17.95%)
Incidence rate	25.3 per 1000 person-years	45.1 per 1000 person-years
Time to diagnosis, years, median (IQR)	1.44 (IQR 0.53–3.11)	2.91 (IQR 1.08–5.42)
Comparative risk for secondary disease diagnosis^a^	Reference group	HR 1.31 (95% CI 1.22–1.40)

^a^Model was adjusted for sex, obesity, current smoker, and variables captured at date of primary disease diagnosed (age, hypertension, hyperlipidaemia, diabetes, MI, COPD stroke, valvular disease, and CKD).

### Heart failure cohort: incidence and risk factors for atrial fibrillation

Among the 10 379 patients with a primary diagnosis of HF, 1863 (17.95%) developed comorbid AF in a median 2.91 (IQR 1.08–5.42) years since diagnosis of the primary disease of HF (45.1 per 1000 person-years; *Table 2*). Independent risk factors for development of AF in patients with HF were older age, male sex, obesity, hypertension, and stroke (*Figure 2B*; *P* < .05 for all).

### Comparative risk for the development of a secondary disease

Compared with patients without AF, patients with prevalent AF had an 11.65-fold higher crude risk of developing HF (45.1 vs 3.87 per 1000 person-years; *Table 2*). After multivariable adjustment, prevalent AF was associated with 3.15 times the risk of new-onset HF (aHR 3.15 95% CI 3.00–3.31; Supplementary Table S3). Similarly, patients with prevalent HF, compared with no HF, had an 11.15-fold increased crude risk of developing AF (25.3 vs 2.27 per 1000 person-years; *Table 2*). However, the risk of AF in HF patients was attenuated after multivariable adjustment (aHR 2.75 95% CI 2.62–2.90); Supplementary Table S3).

**Figure 3:**
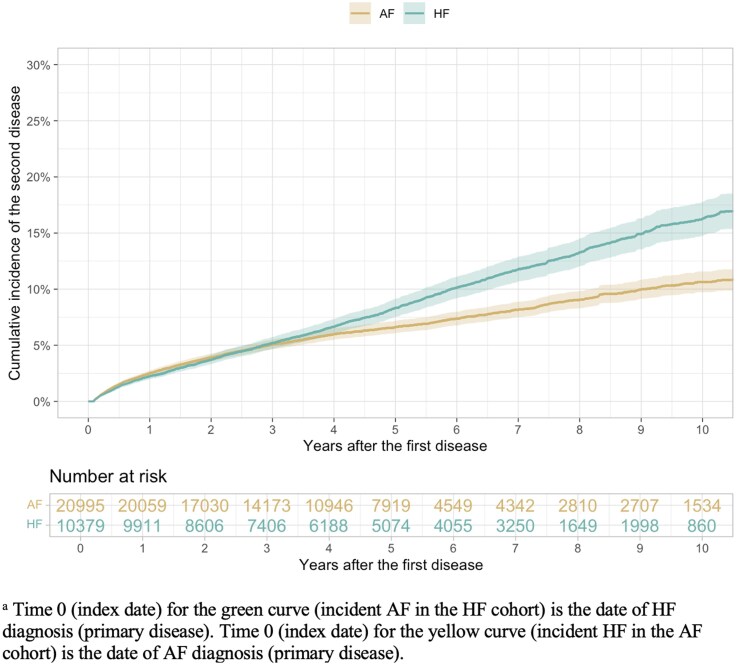
Cumulative incidence curves for the development of the secondary disease

In the comparison of the crude incidence rate for the development of the second disease, the incidence of AF in HF patients is almost double the incidence rate for HF development among AF patients (45.1 vs 25.3 per 1000 person-years, respectively; *Table 2*). As such, the cumulative incidence of AF is approximately 20% among HF patients compared with a 12% incidence of HF among AF patients over a median follow-up of 13 years since the date of primary disease diagnosis (*Figure 3*). HF patients had a longer median time to develop comorbid AF than AF patients who later developed HF (3 vs 1.5 years, respectively; crude). After multivariable adjustment and accounting for the competing risk of death, a direct comparison of the risk for the secondary disease showed that HF patients had a 31% higher risk of AF compared with the risk of HF among AF patients (aHR 1.31, 95% CI 1.22–1.40; *Table 2*; Supplementary Table S4).

### Incidence and risk of all-cause mortality

The crude incidence rate of all-cause mortality was doubled among AF patients who developed incident HF compared with AF patients who did not (55.0 vs 27.9 per 1000 person-years, respectively; *P* < .001 *Table 3*; Supplementary Figures S1 and S2*A*). After multivariable adjustment, the risk of all-cause mortality was almost four times higher in AF patients with incident HF than in patients with AF only (aHR 3.82, 95% CI 3.45–4.24; *Table 3*; Supplementary Table S5). Heterogeneity in effect was only detected for age (*P* for interaction <.001; *Table 3*), with an elevated risk of all-cause mortality detected in AF patients aged <65 years and with incident HF (HR 4.97 95% CI 3.69–6.68; Supplementary Figures S3 and S4).

**Table 3:** Risk of all-cause mortality in disease-specific cohorts

	AF cohort (*N* = 20 995)		HF cohort (*N* = 10 379)	
	AF only (*n* = 18 725)	Incident HF (*n* = 2270)	HF only (*n* = 8516)	Incident AF (*n* = 1863)
Incidence of death, *n* (%)	2365 (12.63%)	678 (29.86%)	3354 (39.38%)	637 (34.19%)
Mortality rate (per 1000 person-years)	27.9 per 1000 person-years	55.0 per 1000 person-years	96.6 per 1000 person-years	50.4 per 1000 person-years
Mortality risk (within cohort)^a^	Reference	HR 3.82 (95% CI 3.45–4.24) Age interaction: *P* < .001 Sex interaction: *P* = .662	Reference	HR 2.31 (95% CI 2.09–2.55) Age interaction: *P* = .301 Sex interaction: *P* = .808
Mortality risk with secondary disease diagnosis (between cohort)^b^		Reference		HR 0.95 (95% CI 0.85–1.07)

^a^Adjusted for age at diagnosis for the primary disease, sex, obesity, current smoker, and time-updated comorbidities.

^b^Adjusted for age at diagnosis for the primary disease, sex, obesity, current smoker, and time-updated comorbidities and days elapsed between the diagnosis of the primary and secondary disease.

Comparatively, the crude mortality rate was also higher in HF patients with incident AF (96.6 per 1000 person-years) than in patients with HF only (50.4 per 1000 person-years; *P* < .001 *Table 3*; Supplementary Figures S1 and S2*B*). After multivariable adjustment, AF was associated with >2 times higher risk of all-cause mortality among HF patients (aHR 2.31, 95% CI 2.09–2.55; *Table 3*; Supplementary Table S5).

**Figure 4:**
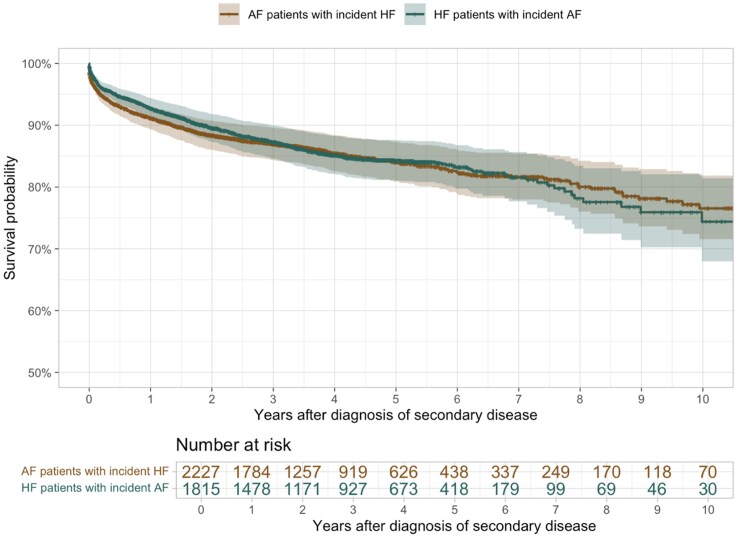
Kaplan–Meier curve comparing the risk of mortality in atrial fibrillation patients with incident heart failure vs heart failure patients with incident atrial fibrillation

In a direct comparison between patients with AF (primary) and incident HF (secondary) to patients with HF (primary) and incident AF (secondary), no statistically significant difference was detected for the risk of all-cause mortality (aHR 0.95, 95% CI 0.85–1.07; *Table 3* and *Figure 4*; Supplementary Table S6).

## Discussion

In a large community-based cohort (*N* = 31 374), we demonstrated that (i) patients with AF or HF had a three-fold increased risk for developing the other comorbid condition, compared with no pre-existing disease; (ii) HF patients had a 31% higher risk of subsequent AF, compared with the risk of AF patients developing HF; and (iii) although presence of both diseases conferred a two- to four-times increased mortality risk, there was no statistically significant difference based on which disease was diagnosed first (*Graphical Abstract*). To our knowledge, this is the largest contemporary study to directly compare the impact of disease temporality on the long-term all-cause mortality risk in patients with both AF and HF.

### Higher risk of developing atrial fibrillation in heart failure and vice versa

The present study demonstrated that patients with either AF or HF had a three-fold increased risk of developing the other disease, compared with participants with neither disease. The crude eleven-fold association attenuated to a three-fold increased risk after adjusting for confounders, reflecting the excess risk of developing the secondary disease following a primary disease diagnosis. In the ARIC cohort, participants with HF had a six- to seven-fold higher risk for prevalent AF and two- to five-fold higher risk for incident AF.^18^ Similarly, the Olmsted study reported an increased risk for development of HF in those with concurrent AF, compared with no AF. Olmsted and Framingham reported slightly higher rates of HF diagnosis in AF patients, at 20% and 33%, respectively.^19,20^ The present study reports lower incidence rates, which may reflect the more contemporaneous cohort, effect of newer treatments for AF and HF, differences in the populations, as well as possible underreporting of HF in the UK Biobank.

Both AF and HF patients had similar independent risk factors associated with secondary disease development, indicating that both diseases share traditional cardiovascular risk factors. Hypertension is a major contributor of incident AF.^21^ Moreover, hypertension, obesity, diabetes, and smoking have been identified as modifiable risk factors accounting for 52% of incident HF, with a greater contribution of 62% in women with AF.^22,23^ Our finding that smoking and a history of a myocardial infarction were associated with a lower risk of incident HF was unexpected but might be caused by multicollinearity or collider bias.

### Heart failure patients at higher risk of atrial fibrillation, compared with the opposite

AF and HF are well-established risk factors for one another, with ‘HF promoting AF and AF aggravating HF’.^24^ However, the present study directly compared the elevated magnitude of risk for the development of the secondary disease and showed that HF patients had a 31% higher risk to develop AF than the risk to develop HF in AF patients. Although AF patients may have a lower risk of developing HF, HF is the primary consequence of AF conferring a greater risk than stroke or mortality, so secondary prevention is essential.

HF patients had a longer median time to AF diagnosis (3 vs 1.5 years, *P* < .001), compared with AF patients developing HF. This suggests a shorter time window for the prevention of developing HF in AF patients. The Olmsted study also identified a 3-year window for HF diagnosis among AF patients.^20^ Results should be interpreted with caution. Time to second disease diagnosis is a crude measure and does not account for the competing risk of death and different clinical profiles. It is plausible that HF patients at a high risk of developing AF may die prior to AF diagnoses. Therefore, only relatively healthy HF patients survive long enough to develop AF and may contribute to the longer time to second disease diagnosis.

### Temporality and mortality risk

Patients with both AF and HF had a similar long-term risk of all-cause mortality, irrespective of which disease was diagnosed first. Few previous studies have investigated the impact of temporality of disease diagnoses on mortality risk.^25,26^ In an analysis of the Danish national registries (*N* = 49 042), AF patients with incident HF had an 8% increased risk of all-cause mortality (aHR 1.08, 95% CI 1.04–1.31). Similarly, HF patients with incident AF also had an increased risk of all-cause mortality when compared with the reference group of concurrent AF and HF (aHR 1.26, 95% CI 1.22–1.30).^25^ Chamberlain *et al.* (*N* = 1664) reported that HF patients with incident AF had more than double the risk of mortality (aHR 2.22, 95% CI 1.93–2.57), whereas those with prevalent AF had a lower risk (aHR 1.29, 95% CI 1.13–1.46), compared with no AF.^26^ However, unlike the present study, no direct comparison of mortality risk between AF patients with incident HF vs HF patients with incident AF was conducted due to the non-collapsibility of hazard ratios.^27^ The lack of difference in mortality risk may reflect the substantial mortality risk inherent to HF, rather than attributable to AF. Future studies are warranted to determine the effect of temporality on treatment efficacy and effectiveness.

Although disease order did not impact the risk of death, the coexistence of AF and HF conferred a higher mortality risk compared with either disease alone. AF patients who developed HF had nearly four-fold increase in mortality risk, while HF patients who developed AF faced a 2.3-fold higher risk. These results are consistent with other population-level studies and subgroup analyses of randomized clinical trials.^28–33^ In the Framingham Heart study, both prevalent and incident HF were associated with poorer survival in AF patients [incident HF; aHR 2.31 (HFpEF), aHR 2.36 (HFrEF)].^4^ Similarly, incident AF in HF across all ejection fraction categories was associated with an increased mortality risk.^4,28^ In a pooled meta-analysis of 22 studies (*N* = 248 323), the coexistence of AF and HF was shown to increase mortality risk by 13% (pooled HR 1.13, 95% CI 1.07–1.21), compared with HF alone.^34^

### Clinical implications

The present study shows that patients with new-onset AF are at very high risk to develop HF in the subsequent years. And vice versa, patients who developed HF have a substantially increased risk to develop AF. In addition, the combination of both diseases confers a much higher risk of mortality than either condition alone. Although causality cannot be assessed from these data, they nevertheless emphasize the opportunity and importance to prevent the onset of HF in patients with newly developed AF and to prevent the onset of AF in those who were just diagnosed with HF.

Several guideline-directed medical therapies have shown to reduce the risk of new-onset AF in patients with HF.^35^ Therefore, optimizing guideline-directed medical therapy is essential, not only to improve morbidity and mortality but also to prevent AF onset and progression. Yet, despite treatment advancements, this study and others have shown that the incidence of AF after HF and vice versa has not declined,^20^ highlighting the need for established and novel secondary prevention strategies. There are currently no evidence-based pharmacological therapies to prevent the development HF in patients with pre-existing AF. However, there is evidence that catheter ablation of AF might reduce the risk of clinical endpoints, including HF hospitalizations. The CASTLE-AF trial randomized patients with symptomatic AF, LVEF ≤ 35%, ICD/CRT-D, and optimal HF therapy to catheter ablation vs guideline-directed drug therapy and showed that ablation reduced the composite of all-cause death or HF hospitalization (28.5% vs 44.6% over ≈38 months).^36^ Furthermore, findings from the CASTLE-HTx trial demonstrated that management of symptomatic AF in advanced HF populations may also improve outcomes.^37^ Several methodological limitations (unblinded design, early stopping, event handling, and loss to follow-up) should be taken into consideration for the clinical application of catheter ablation in patients with AF and HF.

### Limitations

The results should be interpreted in the context of some limitations. Despite the use of population-level data and careful methodological considerations for immortal time bias, time-varying confounding, and direct comparison of hazard ratios, the present study was observational, and causation was not determined. Enrolment in the UK Biobank was voluntary; however, the population primarily consisted of Caucasians (95%), limiting generalizability. Although validated, AF and HF diagnoses were based on hospital and self-reported data. This method may lead to misclassification bias. The timing of diagnosis captured by ICD codes may not precisely correspond to disease onset, limiting the accuracy of temporal analyses. Echocardiographic data at time of HF diagnosis was not available; therefore, HFrEF and HFpEF could not be identified. Finally, although many cardiovascular medications, particularly HF medications, were incorporated as time-varying confounders, catheter ablation and antiarrhythmic medications were not captured, potentially resulting in residual confounding.

## Conclusion

AF and HF strongly predispose to each other, with HF conferring a higher relative risk for incident AF. The coexistence of both diseases substantially increases mortality regardless of which disease developed first, emphasizing the need for strategies to prevent the development of HF in patients with AF and vice versa.

## Supplementary Material

xvag152_Supplementary_Data

## Declarations

### Data Availability

The data underlying this article were provided by the UK Biobank under licence. Data will be shared on request to the corresponding author with permission of the UK Biobank.

### Pre-registered Clinical Trial Number

None supplied.
