# Supplementary material for: Development of atrial fibrillation in patients with heart failure and vice versa: incidence, risk factors, and their impact on survival
Source: ESC Heart Fail. 2026 May 28;13(4):xvag152. doi: 10.1093/eschf/xvag152 (PMC13475117; doi:10.1093/eschf/xvag152)
Supplement: Supplementary file 1 [file xvag152_supplementary_data.docx]

**Supplementary material**

**Supplementary Table 1:** ICD codes for diagnoses

|  | **ICD9** | **ICD10** | **OPCS4** | **Self-reported** ^a^ |
| --- | --- | --- | --- | --- |
| **Heart failure** | 428 | I50, I110, I130, I132, Z941, T862 | K02 | 20002(1076),  20004(1098) |
| **Myocardial infarction** | 410, 412 | I21, I22, I23, I252 |  | 6150(1) |
| **Atrial fibrillation** | 4273 | I48 | K621, K622, K623 | 20002 (1471), 20002 (1483) |
| **DM2** | 25000, 25002, 25010, 25012, 25020, 25022, 25030, 25032, 25040, 25042, 25050, 25052, 25060, 25062, 25070, 25072, 25080, 25082, 25090, 25092 | E11 |  | 20002(1223) |
| **Hyperlipidaemia** | 272 | E78 |  | 20002(1473), 20003 |
| **Hypertension** | 402, 403, 404, 405 | I11, I12, I13, I15, O10 |  | 20002 (1065, 1072), 6150(4), 6177(2), 6153(2), 20003 |
| **COPD** | 492, 496 | J43, J44 |  | 20002 (1112, 1113,1472) |
| **Valvular disease** | 394, 395, 396, 4240, 4241 | I05, I06, I07, I08, I34, I35, I36, I390, I391, I394, I398 |  | 1078,1488,1489,1490,1584,1585,1586,1587 |
| **Stroke** | 430, 431, 432, 433, 434, 436 | I60, I61, I629, I63, I64, I678, I690, I693, G951, H341, H342, S066 | A052, A053, A054, L351, L353, L343 | 6150(3),4056 20002(1081,1491,1583,1086) |
| **Chronic kidney disease** | 585, 586, 588 | I12, I13, N18, T861, Z940 | M01, M084, M172, M174, M178, M179, X401, X402, X403, X404, X405, X406 | 20002 (1192,1193,1194) |

^a^ Including touchscreen medication

**Supplementary Table 2.** Baseline characteristics of AF patients with incident HF versus HF patients with incident AF.

| **Patient characteristics^a,b^** | **AF with incident HF**  **(N= 2270)** | **HF with incident AF**  **(N= 1863)** | **P value** |
| --- | --- | --- | --- |
| Age, years  median [IQR] | 71.39 [67.31, 75.24] | 69.83 [65.36, 73.51] | <0.001 |
| Female | 816 (35.9) | 615 (33.0) | 0.052 |
| Caucasian | 2189 (97.2) | 1750 (94.7)* | <0.001 |
| BMI, kg/m^2^  (mean ± SD) | 30.08 (5.96) | 30.85 (5.99)* | <0.001 |
| Obesity | 956 (42.6) | 901 (49.2)* | <0.001 |
| Waist to hip ratio  (mean ± SD) | 0.92 (0.09) | 0.94 (0.09)* | <0.001 |
| Current smoker | 286 (12.7) | 260 (14.1) | 0.206 |
| Current drinker | 2041 (90.4) | 1626 (87.8)* | 0.009 |
| Socioeconomic status | | * | 0.002 |
| Low | 582 (25.6) | 564 (30.3) |  |
| Median | 625 (27.5) | 509 (27.4) |  |
| High | 1063 (46.8) | 787 (42.3) |  |
| ***Medical history*** |  |  |  |
| Diabetes Mellitus II | 472 (20.8) | 525 (28.2)* | <0.001 |
| Hypertension | 1737 (76.5) | 1532 (82.2)* | <0.001 |
| Hyperlipidemia | 1354 (59.6) | 1230 (66.0)* | <0.001 |
| MI | 383 (16.9) | 613 (32.9)* | <0.001 |
| COPD | 365 (16.1) | 352 (18.9)* | 0.019 |
| Stroke | 264 (11.6) | 251 (13.5) | 0.082 |
| Valvular disease | 457 (20.1) | 585 (31.4)* | <0.001 |
| CKD | 269 (11.9) | 296 (15.9)* | <0.001 |
| ***Medication*** |  |  |  |
| ACEi | 589 (26.3) | 605 (32.8)* | <0.001 |
| ARB | 6 (0.3) | 2 (0.1) | 0.431 |
| Beta-blockers | 356 (15.7) | 317 (17.1) | 0.253 |
| Calcium channel blockers | 520 (23.1) | 475 (25.6) | 0.072 |
| Lipid-lowering drug | 541 (23.8) | 354 (19.0)* | <0.001 |

^a^ Frequencies and percentages (n(%)) are presented, unless otherwise specified.

^b^All patient characteristics were reported at baseline or cohort entry. Baseline was the date of diagnosis of the primary disease, except for BMI, obesity, waist to hip ratio, current smoker, current drinker, socioeconomic status and medication, this was at cohort entry.

*Depicts a statistically significant (p<0.05) comparison

^c^Abbreviations: ACEi, angiotensin-converting enzyme inhibitor; ARB, angiotensin-receptor blocker; BMI, body mass index; MI, myocardial infarction; COPD, chronic obstructive pulmonary disease; CKD, chronic kidney disease.

**Supplementary Table 3.** Fine-Gray model for the risk of secondary disease in patients with primary disease, compared to no pre-existing disease.

| **Incident AF (secondary)**  **N=24,960** | **No pre-existing disease**  **N=483,072** | **HF (primary)**  **N=10,379** | **P-value** |
| --- | --- | --- | --- |
|  |  | **HR (95%CI)** |  |
| Model 1 | Reference | 6.224 (5.944 - 6.517)* | <0.001 |
| Model 2^a^ | Reference | 3.653 (3.484 - 3.830)* | <0.001 |
| Model 3^bc^ | Reference | 2.753 (2.617 - 2.896)* | <0.001 |
| **Incident HF (secondary)**  **N=14,751** | **No pre-existing disease**  **N=472,456** | **AF (primary)**  **N=20,995** | **P-value** |
| Model 1 | Reference | 6.627 (6.335 - 6.932)* | <0.001 |
| Model 2^a^ | Reference | 3.973 (3.791 - 4.164)* | <0.001 |
| Model 3^bc^ | Reference | 3.147 (2.995 - 3.306)* | <0.001 |

^a^ Adjusted for age at baseline and sex

^b^ Adjusted for age at baseline, sex, obesity, current smoker, hypertension, hyperlipidaemia, diabetes, MI, COPD stroke, valvular disease and CKD.

^c^Risk of secondary disease in patients who have a primary disease, compared to no pre-existing disease.

**Supplementary Table 4:** Cox regression for comparing the incidence of the secondary disease in HF patients with incident AF and AF with incident HF.

|  | **AF with**  **incident HF**  **(N=2270)** | **HF with**  **incident AF**  **(N=1863)** |  |
| --- | --- | --- | --- |
|  |  | **HR (95%CI)** | **P-value** |
| **Model 1** | Reference | 1.49 (1.41-1.58)* | <0.001 |
| **Model 2**^a^ | Reference | 1.54 (1.45-1.64)* | <0.001 |
| **Model 3**^b^ | Reference | 1.31 (1.22-1.40)* | <0.001 |

^a^ Adjusted for age at diagnosis for the primary disease and sex

^b^Adjusted for age at diagnosis for the primary disease, sex, obesity, current smoker and time-updated comorbidities.

**Supplementary Table 5:** Time-dependent multivariable Cox regression for all-cause mortality.

|  | **AF cohort (N=20995)** | |  |
| --- | --- | --- | --- |
|  | **No incident HF (N=18725)** | **Incident HF** ^a^  **(N=2270)** |  |
|  |  | **HR (95% CI)** | **p-value** |
| **Model 1** | Reference | 5.82 (5.29-6.41) | <0.001 |
| **Model 2** ^b^ | Reference | 5.34 (4.84-5.88) | <0.001 |
| **Model 3** ^c^ | Reference | 3.82 (3.45-4.24)  Age interaction: p<0.002  Sex interaction: p=0.662 | <0.001 |
|  | **HF cohort (N=10379))** | |  |
|  | **No incident AF (N= 8516)** | **Incident AF**^a^  **(N=1863)** |  |
|  |  | **HR (95% CI)** | **p-value** |
| **Model 1** | Reference | 2.95 (2.68 - 3.25) | p<0.001 |
| **Model 2** ^b^ | Reference | 2.75 (2.50 - 3.04) | p<0.001 |
| **Model 3** ^c^ | Reference | 2.31 (2.09 - 2.55)  Age interaction: p=0.301  Sex interaction: p=0.808 | p<0.001 |

^a^ Comorbid condition as time-dependent variable

^b^ Adjusted for age at diagnosis for the primary disease and sex

^c^ Adjusted for age at diagnosis for the primary disease, sex, obesity, current smoker and time-updated comorbidities.

**Supplementary Table 6:** Time-dependent multivariable Cox regression for comparing mortality in HF with incident AF and AF with incident HF.

|  | **AF with incident HF**  **(N=2270)** | **HF with incident AF**  **(N=1863)** |  |
| --- | --- | --- | --- |
|  |  | **HR (95%CI)** | **p-value** |
| **Model 1** | Reference | 1.10 (0.99-1.23) | 0.074 |
| **Model 2** ^a^ | Reference | 1.11 (0.99-1.24) | 0.063 |
| **Model 3** ^b^ | Reference | 1.12 (1.00-1.26) | 0.042 |
| **Model 4** ^c^ | Reference | 0.93 (0.83-1.04) | 0.220 |
| **Model 5** ^d^ | Reference | 0.95 (0.85-1.07) | 0.413 |

^a^ Adjusted for age at diagnosis of the secondary disease and sex

^b^ Adjusted for age at at diagnosis of the secondary disease, sex, and days elapsed between primary and secondary diagnosis

^c^ Adjusted for age at diagnosis for the secondary disease, sex, obesity, current smoker and time-updated comorbidities.

^d^ Adjusted for age at diagnosis for the secondary disease, sex, obesity, current smoker and time-updated comorbidities, and days elapsed between primary and secondary diagnosis

**Supplementary figure 1**: Adjusted Kaplan Meier curves for the risk of all-cause mortality

A) AF cohort

B) HF cohort

^d^ Adjusted for age at diagnosis for the primary disease, sex, obesity, current smoker and comorbidities.

**Supplementary figure 2.** Adjusted Kaplan Meier curves for the risk of all-cause mortality with and without a secondary disease diagnosis

A) Incident HF versus no incident HF in the AF cohort

B) Incident AF versus no incident AF in the HF cohort

**Supplementary figure 3.** Subgroup analyses stratified by age categories and sex

A) Incident HF in the AF cohort

*B)* Incident AF in the HF cohort

**Supplementary figure 4:** Spline by age for the prognostic impact of incident HF in AF cohort
